# Cardiovascular Reflexes - Vagus as the Key Player

**DOI:** 10.2174/011573403X284831240408053528

**Published:** 2024-04-29

**Authors:** Shantala S. Herlekar, Ashwini R. Doyizode, Savitri P. Siddangoudra, V. Anupama

**Affiliations:** 1 Department of Physiology, J N Medical College, Belagavi, Karnataka, India;; 2 Department of Physiology, SDM College of Medical Sciences and Hospital, Dharwad, Karnataka, India;; 3 Department of Physiology, KAHERs JGMM Medical College, Hubballi, Karnataka, India

**Keywords:** Vagus, Sino-aortic nerves, cardio-vascular reflexes, baroreceptor resetting, mareys reflex, reverse bainbridege reflex

## Abstract

The cardiac and vascular systems work in coordination by activating various reflex mechanisms based on the body’s needs. These may be during physiological variations or pathophysiological changes seen in disease conditions of varying degrees of severity. This article intends to explain various reflexes involved in the homeostasis of the cardiovascular system and the role of vagus as the key component in all these reflexes. The article also explains the components of the reflex arc, the stimulus and response, and the role of reflex in a few diseases. This article describes 22 different cardiovascular reflexes in detail.

## 1 INTRODUCTION

### 1.1 The VAGUS Nerve (Cranial Nerve 10)

The longest cranial nerve, the VAGUS Nerve, has both efferent (sensorial and motor) and afferent (motor) fibers. It regulates the functions of almost all the visceras, including tongue, pharynx, heart, lung, GIT and more [1-10].

Vagus originates from medulla and passes through the superior and inferior ganglion as it passes through the jugular foramen. It has the following nuclei and functions:

- *Nucleus Ambigus*: Which helps to mediate swallowing and phonation.
- *Dorsal motor nucleus*: Which supplies the involuntary muscles of the cardiovascular system, lungs, esophagus and GIT.
- *Through the superior ganglion*: Innervates the external ear and tympanic membrane.
- *Through inferior ganglion*: Supplies carotid and aortic bodies, also supplies fibers for taste sensation.
- *Nucleus tractus solitarius*: Source of vagal efferent.

**Fig. (1):**
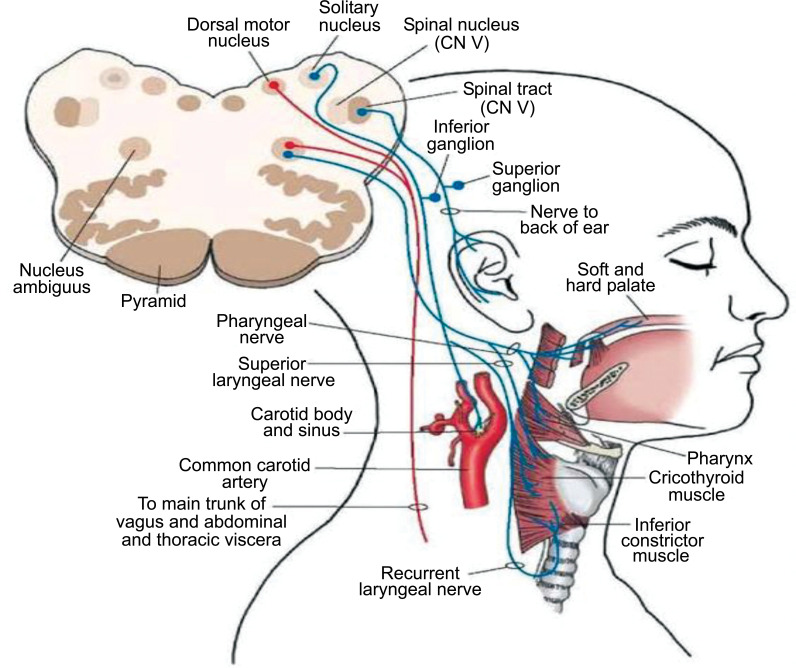
The Nuclei, ganglias and Distribution of the Vagus Nerve including autonomic components. Note the Nucleus ambigus, dorsal motor nucleus and NTS within medulla. Also the superior and inferior ganglia of vagus [8, 9].

Vagus has multiple functions. Vagal stimulation causes cough, apnea, and controls the heart by producing bradycardia and hypotension, regulates deglutition, coordinates speech articulation, *etc*. (Fig. **1**). Therapeutic uses include vagus stimulation to treat epilepsy and depression, and also the ongoing research points towards the treatment of obesity, myocardial infaraction, supraventricular tachycardia, *etc*. Lateral medullary syndrome affects nucleus ambigus.

### 1.2 Sino-aortic Nerves

Nerves supplying carotid and aortic body (chemoreceptors) and carotid sinus and aortic arch (baroreceptors) *i.e*., carotid sinus nerve, also called Herings nerve, are the branches from the glossopharyngeal nerve and vagus nerve. These are also called the buffer nerves, as they prevent rapid alterations of blood pressure [10].

With this brief introduction, let us look at the various reflexes in which the vagus participates, specifically in relation to the cardiovascular system.

## 2 CARDIOVASCULAR REFLEX AND THE ROLE OF VAGUS

### 2.1 Vagal Tone

Heart is under the control of both the components of autonomic nervous system *i.e.* sympathetic and the parasympathetic nerves, parasympathetic (vagus) dominates. Tonic vagal discharge to the heart is called vagal tone. It is an inbuilt reflex and is produced by the glossopharyngeal and vagus nerve. Stimulation of vagi releases the neurotransmitter *acetylcholine* from the vagal endings. Acetylcholine affects the heart in two major ways [2-6].

- Reduces sinus nodal rhythm, that is, the rate at which sinus node discharges will reduce.
- Excitability of the A-V junctional fibers decreases thereby decelerating impulse transmission from atria into the ventricles.

The resting HR of 72 beats/min rises to 150-180 beats/min when both the vagi are cut or blocked. If both sympathetic and parasympathetic are cut, HR is approximately 100 b/min. This confirms that vagal tone is more than sympathetic tone at rest.

### 2.2 Mechanism of the Vagal Effects

Cardiac parasympathetic nerves arise from the cells within the dorsal motor nucleus of vagus and/or Nucleus Ambigus. These vagal fibers proceed as cervico-vagal fibers and, pass through the mediastinum and terminate as the pre ganglionic fibers within the epicardium. Majority of the ganglions lie around the SA node. The right vagus affects SA nodal activity while the left vagus delays AV nodal conduction. Hyperpolarisation of the conductive fibers caused by increased influx of potassium remains the mainstay of action of acetylcholine released by vagal nerve endings. This makes the excitable tissue much less excitable. The “resting” membrane potential shifts from the normal levels of -55 to -60 mv to a considerably more negative value of -65 to -75 mv. Therefore, the inward sodium and calcium movement, which depolarises SA-nodal membrane, takes much longer to reach the threshold potential necessary for excitation. This reduces the rate of rhythmic discharge of these nodal fibers to a large extent.

The cause for the predominant vagal tone on the heart can be explained based on “NERVE-NERVE INTERACTION” between the two divisions of autonomic nervous system called the interneuronal and intracellular mechanisms.

- *Interneuronal mechanism* – explains that a branch of vagus supplying the SA node of the heart, reaches the sympathetic nerve and causes pre-junctional inhibition of sympathetic innervation, thus preventing the release of NE.
- *Intracellular mechanism –* explains that there is competitive inhibition of adenyl cyclase onto which both the vagus activated muscarinic receptor (of SA node) and the NE of sympathetic nervous system acts, the vagus predominates. This is mediated *via* the Gi receptors.

Vagal escape (also called venous reflex) [2, 6]. In animal experiments, mild to moderate vagal excitation slows the HR to nearly one-half normal. The impulses are completely blocked between atria and ventricles, and within SA node when the vagus is stimulated nonstop. But after a while, the heart starts beating again even if vagal stimulation is continued, called “vagal escape”. Here, the heart escapes from the inhibitory influences of vagus. Following mechanisms have been suggested for vagal escape:

- After the heart stops, engorgement of great veins and right atrium inhibits vagal afferents to medulla and increases the sympathetic discharge.
- After the heart stops functioning, BP falls in the aorta causing inhibition of baroreceptor reflex, and chemoreceptors in aortic and carotid bodies are stimulated. These together increase sympathetic discharge.
- Due to a complete block in the conduction of impulses from atria to ventricle (AV nodal block), ventricle escapes from the control of SAN and develops its own rhythm called the idioventricular rhythm (ventricular escape).

### 2.3 Sinus Arrhythmia

During inspiration, HR increases (*sinus tachycardia*); during expiration, HR decreases (*sinus bradycardia*). This is called sinus arrhythmia. Often seen with regular respiration in healthy children, in healthy adults it becomes evident with deep voluntary inspiration and expiration. Atropine abolishes this reflex, showing that the vagus is involved (*Atropine blocks Ach from binding to its receptors*). Possible mechanisms suggested for sinus arrhythmia are [7, 11-14]:

- Afferent impulses from the lungs during inspiration reflexly inhibit vagus, which will in turn cause inspiratory tachycardia.
- Inspiration > increases negative intrathorasic pressure > which increases venous return > causing engorgement of right heart > leads to interventricular septum pushed to left > less blood flow to left ventricular > less left ventricular ejection fraction > BP within the aorta reduces > baroreceptor reflex is abolished > reduced vagal tone and increased sympathetic discharge >heart rate increases.
- Impulses from respiratory centers may irradiate to neighboring cardiac centers within the medulla during inspiration, inhibiting vagal discharge from medullary centres (This is also called central irradiation).

Of the 3 mechanisms mentioned, central irradiation theory is wildly accepted.

Afferent: Central pacemaker for respiratory

Centre: Nucleus ambiguus

Efferent: Through the cardiac ganglion, to vagus.

**Fig. (2):**
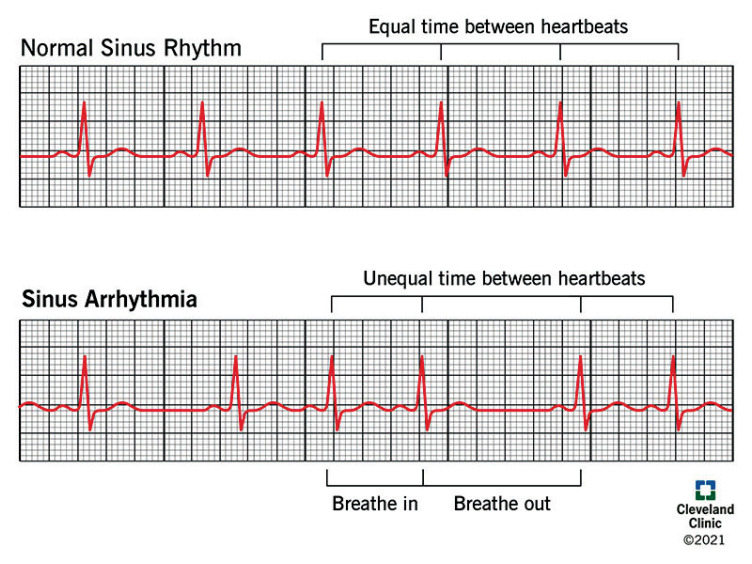
Active ECG changes comparing changes seen during normal respiration and deep breathing. The heart rate shown by measuring RR interval is compared in both cases. RR interval is regular in normal sinus rhythm, while it shows tachycardia in inspiration and bradycardia during expiration in sinus arrhythmia [15].

Effect: Physiological increase in heart rate during inspiration (Fig. **2**).

### 2.4 Baroreceptor Reflex

Whenever there is a disturbance in the circulatory homeostasis, the baroreceptor plays a significant role in stabilizing the perfusion pressure. Baroreceptors are mechanoreceptors which, *via* their sympathetic and vagal connections, regulate both cardiac output and peripheral resistance, and maintains blood pressure homeostasis. There are 2 types of baroreceptors, called high-pressure and low-pressure receptors [16-21].

Receptors located within the carotid sinus and aortic arch are termed High-pressure receptors. These high-pressure baroreceptors in-turn have 2 types of fibers, dynamic and tonic, and are innervated with myelinated Type A fibers in dynamic and unmyelinated Type C fibers in static. These high-pressure receptors respond progressively and more rapidly to mean arterial blood pressure (MAP) changes ranging between 50-160 mmHg. It responds to both increase and decrease MAP hence is also called “pressure-buffer system”. At resting blood pressure, baroreceptors respond to stable MAP by displaying a tonic excitatory transmission to the nucleus tractus solitarious (NTS) *via* the vagus and glossopharyngeal nerve (which are also called sino-aortic nerves). From NTS, the tonic sympathetic flow reaches the peripheral blood vessels. When the MAP decreases compared to resting MAP, baroreceptor discharge decreases, decreasing tonic excitation of NTS, and leading to the activation of the sympathetic supply to the heart and peripheral vessels. This results in BP to be normal. When MAP increases above the resting MAP, NTS is stimulated, tonic sympathetic flow decreases to blood vessels causing vasodilatory effect.

The low-pressure receptors, also called the cardiopulmonary receptors, are located within the atrias, ventricles and pulmonary vasculature. These are activated by either changes within atrial and ventricular wall tension or wall stretch causing the following effects:

- Reduces sympathetic flow to the kidney, which reduces renal blood flow and urine output.
- Increases sympathetic flow to SA node, which increases heart rate and cardiac output.
- *Via* vagal effects on the medulla, reduces angiotensin, aldosterone and vasopressin release.

In low volume conditions, circulatory and renal changes result in retention of salt and water. Low pressure receptors are also known to regulate cerebral circulation and are active during dynamic exercise and body position changes.

Baroreceptor dysfunction plays a significant role in symptoms associated with orthostatic hypotension, refractory hypertension, carotid sinus syndrome, syncope with shaving, carotid sinus massage in supraventricular tachycardia, refractory heart failure, insulin resistance and squamous cell carcinoma induced orthostatic hypotension [22].

### 2.5 Baroreceptor Resetting

**Fig. (3):**
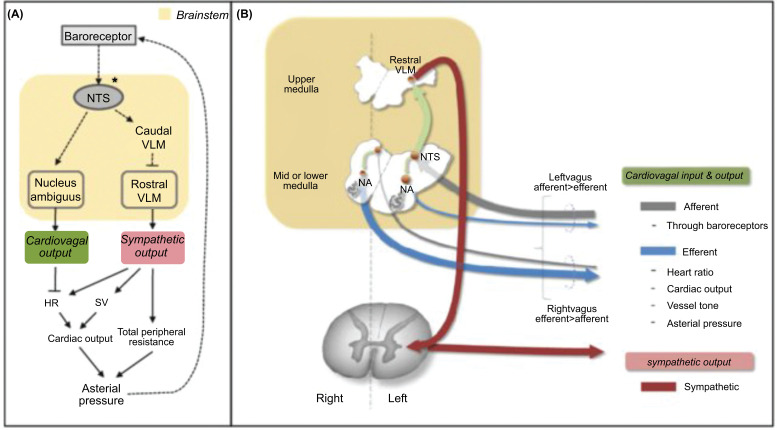
Components of the baroreceptor reflex involve nucleus tractus solitarius (NTS) and the ventral medulla. It acts as a feedback mechanism to minimize fluctuations in arterial BP. Part b shows afferent and efferent vagal pathways [21].

Baroreceptors are important for the regulation of minute to minute or beat to beat variations in BP (specifically MAP and PP). It regulates BP by adjusting heart rate, modifies the vasomotor centre discharge and controls the secretion of hormones like epinephrine and norepinephrine. But this action lasts for only a few hours to a few days (Fig. **3**) [18, 19, 23-26].

Fluctuations of arterial blood pressure activate the baro-reflex as a part of a homeostatic mechanism that neutralizes these fluctuations. Physical activity like exercise causes a rise in heart rate, arterial blood pressure, cardiac output and sympathetic activity. This is a physiological response to exercise and is seen to occur with an intact and functional baroreceptor. This varied response of baro-reflex has been studied using variable pressure neck collar in humans and in animal experiments. Studies suggested that compared to resting levels, during physical exercise, baroreceptors retuned to fire normally, even at an upper range of arterial pressure. The gain of the entire system remains unaffected. Afferents generated from mechanoreceptors and chemoreceptors of active skeletal muscles, along with the Central command by motor cortex are the 2 factors that are responsible for the baroreceptor resetting seen during exercise. Thus, within physiological limits, baroreceptors response is to be made null and void during exercise as body needs higher blood pressure to maintain homeostasis during exercise.

In chronic hypertension (SBP>135 mm Hg, DBP>90 mm Hg, MAP>110 mm Hg), the baroreceptor readjusts (reset) in 1-2 days to the new elevated levels of blood. In other words, if the arterial blood pressure rises from 100 mm Hg (normal range) to 160 mm Hg (elevated), baroreceptor initially starts firing at a very high rate. The firing rate continues for a few minutes, after which it diminishes considerably; reduction occurs in the next 1 to 2 days. Eventually, after this time, the rate of firing reverts to near normal despite the elevated mean arterial pressure at 160 mm Hg. In opposition, when the arterial blood pressure dips to a considerably low level, initially baroreceptors generate no impulses, but eventually, over 1 to 2 days, the rate of baroreceptor activation returns to normal firing level despite the persistent low blood pressure. Thus baroreceptors have no part in long term regulation of arterial blood pressure.

**Fig. (4):**
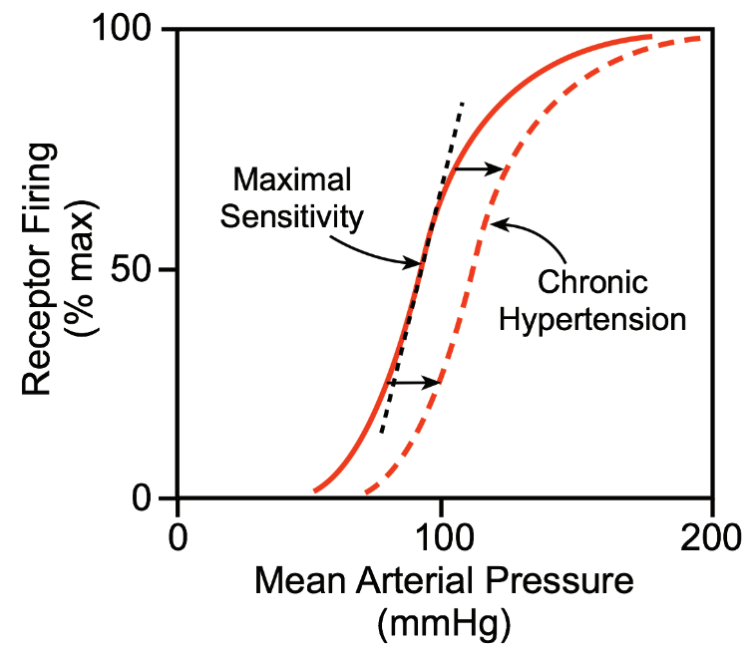
Shows firing rate of carotid sinus. Solid lines indicates the firing rate at normal MAP while dashed lines, the firing rate of carotid sinus is established at elevated MAP [26].

However, recent research gives evidence that the baroreceptors do not entirely reset and may have a part in long-term blood pressure regulation, chiefly by their influence on the sympathetic nerve activity of the renal system. With long term increase in blood pressure, the baroreceptor reflexes may cause a reduction in renal sympathetic outflow, promoting increased elimination of salt and water by renal tubules. This gradually helps in reducing blood volume, which reestablishes arterial blood pressure to a normal level. Thus, long-term control of mean arterial blood pressure by the baroreflex needs additional interaction with renal–body fluid–pressure regulatory system (alongside its association with nervous and hormonal control systems) (Fig. **4**).

### 2.6 CNS Ischemic Response

Neural regulation of blood pressure is attained by reflexes that are generated within the baroreceptors system, the chemoreceptors, and reflexes generated within the low-pressure receptors system. All of these are situated in the peripheral circulation outside the central nervous system. However, when there is a severe reduction in blood flow to medullary vasomotor center, it leads to nutritional deficiency—that is, cerebral ischemia. This causes direct activation of cardioaccelerator neurons and vasoconstrictor neurons located in the vasomotor center, which respond to the ischemia and they become strongly excited. This further leads to a rise in systemic arterial pressure as high as the heart's capability to pump [27-29].

When blood flow to the vasomotor center is low, carbon dioxide concentration increases excessively within local areas of the brain and is the most potent activator of medullary sympathetic vasomotor areas. Also, other local factors, like lactic acid, may lead to a marked elevation of arterial blood pressure. This rise in arterial pressure in response to cerebral ischemia and accumulated metabolites is known as CNS ischemic response.

The extent of the ischemic effect on vasomotor function is so strong that it can completely occlude some of the peripheral arteries and increase mean arterial pressure for up to 10 minutes, sometimes to as high as 250 mm Hg. As an example, when the sympathetic discharge occurs, the kidneys experience severe arteriolar vasoconstriction, which frequently results in complete cessation of urine production. As a result, among all the sympathetic vasoconstrictor system activators, the CNS ischemia response is one of the strongest.

**Fig. (5):**
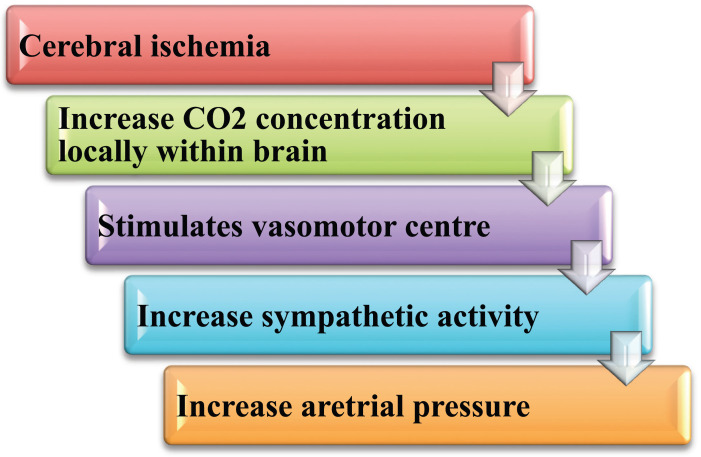
Flow chart showing the various steps which activates CNS Ischemic response.

Even while the CNS ischemia response is strong, it doesn't become noticeable until the arterial pressure drops well below normal, down to 60 mm Hg and lower, and reaches its peak of activation at 15 to 20 mm Hg. As a result, it does not function as a typical arterial pressure regulation mechanism. Rather, it functions primarily as an emergency pressure control mechanism, acting swiftly and forcefully to stop arterial pressure from falling any lower. This comes into action only when the brain blood flow falls perilously close to the point of death. Hence, it is also referred to as the pressure control mechanism's “last ditch stand” (Fig. **5**).

### 2.7 Cushings Reflex

A unique kind of CNS ischemia response known as the “Cushing reaction” is seen when there is an increase in the pressure of the cerebrospinal fluid surrounding the brain in the cranial vault. Cushing's triad consists of widening pulse pressure (increasing systolic, decreasing diastolic), bradycardia, and irregular respirations. This is the physiological response of the nervous system to sudden increases in intracranial pressure (ICP). The normal ICP is measured between 5 and 15 mmHg. Cushings reflex is activated due to the compression of arteries within the brain. There is a cut-off of the cerebral blood supply when the cerebrospinal fluid pressure increases to the same level as the arterial pressure. This starts an ischemia reaction within the central nervous system, which raises blood pressure. Blood flows back into the brain's capillaries to treat brain ischemia once the arterial pressure rises to a level greater than the cerebrospinal fluid pressure. The blood normally reaches a new equilibrium level that is marginally higher than the pressure of the cerebrospinal fluid, which permits blood to start flowing into the brain tissue once more. In the unlikely event that the cerebral arteries are compressed by a high enough level of cerebrospinal fluid pressure, the Cushing reaction serves to shield the brain's essential areas from malnourishment [30-33].

In the initial stages, high ICP increases PCO_2_ retention and activates the sympathetic system. These increase both heart rate and blood pressure. If ICP continues to remain high, probably the elevated BP activates the baroreceptor reflex or, from actual compression of intracranial vagus nerve, causes bradycardia. Also, raised ICP causes compression of brainstem respiratory centres, leading to periods of apnoea.

Afferent: Rostral medullary mechanosensors?

Processor: Rostral ventrolateral medulla

Efferent: Sympathetic afferents to cardia and vascular smooth muscle

**Fig. (6):**
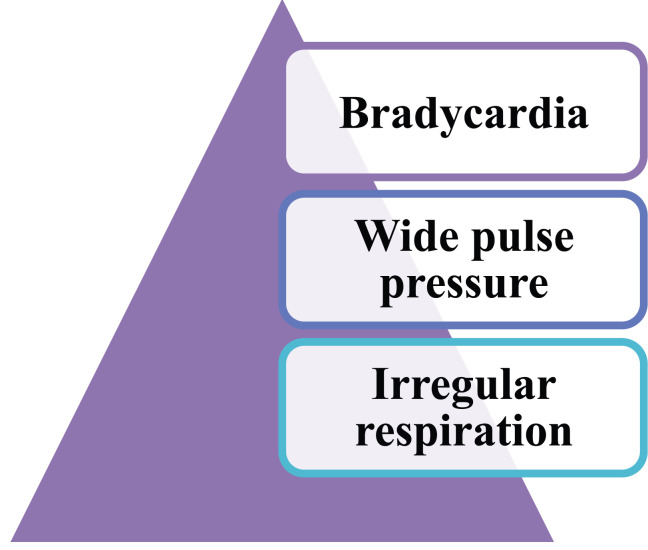
Represents the triad of symptoms seen in Cushings reflex.

Effect: Hypertension and baroreflex-mediated bradycardia (Fig. **6**).

### 2.8 Bainbridge Reflex (also called Atrial Reflex or Cardio-accelerator Reflex)

Low-pressure receptors are stretch receptors found in the walls of the pulmonary arteries and the atria. These low-pressure sensors are crucial, particularly when it comes to reducing changes in arterial pressure brought on by variations in blood volume. They bear a resemblance to the major systemic artery stretch receptors, also known as baroreceptors (high-pressure receptors). They cannot detect systemic arterial pressure changes but can detect pressure changes in low pressure areas caused by an increase in blood volume. They act in parallel to the baroreceptor reflex; both reflexes together make an efficient system for controlling arterial pressure changes [34-37].

If the initial heart rate is low (<130 beats/min), infusion of normal saline or blood > causes stretching of low-pressure receptors > which activates a nervous reflex called the Bainbridge reflex > passing to the vasomotor center of the brain *via* vagus > then by way of the sympathetic nerves and vagi, back to the heart > finally elevates the heart rate (Tachycardia). The right atrial wall gets stretched and so does sinus node in the wall of the right atrium, both together cause increasing heart rate. Consequently, this reflex aids in preventing blood damming in the pulmonary circulation, atria, and veins. The Bainbridge reflex is an extension of respiratory sinus arrhythmia.

1. Afferent: Vagus (atrial stretch)
2. Processor: NTS (nucleus of the solitary tract) and the caudal ventral medulla
3. Efferent: Vagus nerve and sympathetic chain

**Fig. (7):**
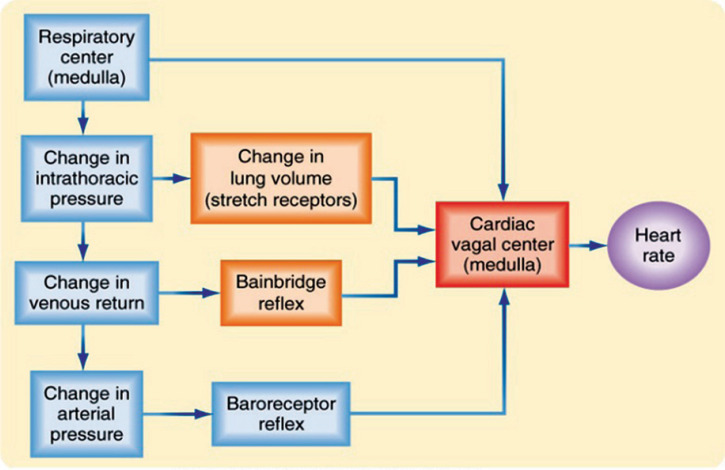
[38] shows 3 cardiovascular reflex mechanisms acting side by side in response to respiratory changes, changes in blood volume and arterial pressure and causes changes in heart rate.

Effect: increased RA pressure produces an increased heart rate;

If the initial heart rate is high (>130 b/min), infusion of normal saline or blood causes bradycardia rather than tachycardia. This reflex is also mediated by vagus (Fig. **7**).

#### 2.8.1 Reverse Bainbridge Reflex

This represents a complete cardiopulmonary reflex, which includes both increase in heart rate seen during hypervolemia and heart rate falls seen during hypovolemia. A drop in the heart rate, which is seen when there is drop in venous return, observed in instances of controlled hypotension, spinal and epidural anesthesia, and severe bleeding, has been attributed to a “reverse” Bainbridge reflex. A reverse Bainbridge reflex would suggest that baseline cardiopulmonary receptor activity is present and that this activity influences the firing rate of the sinoatrial (SA) node in a tonic stimulatory manner. Reduced venous return would therefore result in the unloading (deactivation) of these receptors and cause reflex reduction in heart rate [34, 39-42].

### 2.9 Volume Reflex: Atrial Reflexes That Activate the Kidneys

When there is volume overload, the atrial stretch receptors facilitate the return of blood volume to normal. This is called volume reflex. The afferent is the vagus, while efferents reach the heart and kidneys through sympathetic nerves. It occurs by following mechanisms [43-46]:

- Stretch of the atria brings substantial reflex dilation of the afferent arterioles in the kidneys, causing glomerular capillary pressure to increase. This increases the filtration of fluid in renal tubules.
- Stretched atrias also transmit signals to the hypothalamus to decrease Antidiuretic hormone (ADH) secretion. Antidiuretic hormone in turn diminishes the reabsorption of water within renal tubules.

The combination of these two effects, a rise in glomerular filtration and a fall in fluid reabsorption, increases the kidneys' ability to lose fluid and brings the blood volume back to normal.

c. Stretch of Atria caused by blood volume also releases Atrial Natriuretic Peptide (ANP), a powerful diuretic agent that adds further to the elimination of fluid in the urine and returns the blood volume to normal.

The above 3 reflexes are volume controllers. Excess volume increases cardiac output which increases blood pressure. So these are also called pressure controllers.

### 2.10 Bezold-Jarisch Reflex

Some regions in the heart are sensitive to stretch, chemical changes and certain drugs for *e.g.:* Alveolar juxtacapillary area, atria, ventricles, pulmonary artery and great veins. These are supplied by chemosensitive unmyelinated vagal C fibers. These can be activated by various chemicals like capsaicin, serotonin, phenylbiguanide, and veratridine [47-50].

They produce a depressor reflex with a triad of a) bradycardia; b) hypotension; and c) coronary artery vasodilatation. It also causes momentary apnea and subsequent rapid shallow breathing. These are mediated by parasympathetic stimulation and downregulation of the sympathetic system. This is called the Bezold–Jarisch reflex response and it gets its name after the researcher who first narrated these findings.

Originally, it was only a pharmacologic curiosity, now, it is seen to be activated in many pathophysiological conditions. For example,

- In myocardial ischemia, the release of chemicals from the injured myocardium causes severe bradycardia and hypotension. This is a stubborn complication of MI during treatment protocol.
- During coronary angiography.
- This reflex participates in a defense system that shields people from hazardous chemicals. By preventing the blood from becoming overly toxic from inspired pollutants and aiding in their removal from the body, activation of the cardiopulmonary reflexes may help lower the quantity of pollutants that are absorbed into the blood.

Afferent: Activation of vagus either mechanically or chemically within cardiac chambers.

Processor: Nucleus of the solitary tract

Efferent: Vagus nerve along with sympathetic chain

Effect: Atrial stimulation leading to hypotension and bradycardia.

### 2.11 Vasovagal Syncope (Barcroft-Edholm Reflex)

The Bezold-Jarisch reflex has also been linked to the syndrome of cardiac slowing with hypotension. Prolonged upright posture can cause vasovagal syncope, also known as postural syncope, which is characterized by decreased intracardiac blood volume and blood pooling in the lower extremities. When dehydration is added to this phenomenon, it becomes more pronounced. The carotid sinus baroreceptors, also known as high-pressure receptors, detect the ensuing arterial hypotension. Afferent fibers from these receptors then initiate autonomic signals that raise the cardiac rate and contractility. Low pressure receptors found in the left ventricle wall, on the other hand, react by sending signals that cause paradoxical bradycardia and reduce contractility, which causes abrupt, significant hypotension. In addition, the person feels dizzy and might have a brief period of unconsciousness [51, 52].

Afferent: Hypovolaemia, emotional suffering

Processor: Unknown

Efferent: Vagus nerve along with sympathetic chain

Effect: Systemic vasodilation, hypotension, bradycardia.

## 3 VASOMOTOR TONE

### 3.1 (Heart - Vagal/ Parasympathetic Tone Vessels - Vasomotor / Sympathetic Tone)

Normally, the sympathetic vasoconstrictor nerve fibers throughout the body receive continuous signals from the vasoconstrictor area of the vasomotor center (VMC) in the medulla, which causes the fibers to fire slowly and continuously at a rate of roughly two impulses per second. Sympathetic vasoconstrictor tone is the term for this continuous firing. Vasomotor tone, a state of partial contraction of the blood vessels, is typically maintained by these impulses [2, 4, 6, 53].

When *sympathetic vasoconstrictor tone* is blocked in experimental animals, the vasoconstrictor tone is lost throughout the body, causing a fall in arterial pressure from 100 to 50 mm of Hg.

IMPORTANCE: Neurogenic Shock [54-56]-Shock can ensue without any loss in blood volume. When the sympathetic tone is completely lost, the vascular capacity (capacity within the blood vessels) rises to such an extent that the circulatory system pressure is not enough to sufficiently fill normal blood vessels and maintain circulation. The veins enlarge dramatically as a result. The ensuing state is called neurogenic shock. Why People Get Neurogenic Shock? The common causes are:

- Deep general anesthesia frequently causes the vasomotor center to become sufficiently depressed, leading to vasomotor paralysis and neurogenic shock.
- Spinal anesthesia inhibits the sympathetic nervous system's outflow, particularly when the anaesthetic agent circulates through the entire spinal cord.
- Vasomotor paralysis is frequently caused by damage to the brain, leading to failure of vasomotor centre and neurogenic shock.

### 3.2 Reactive Hyperemia

Blood flow through a tissue usually increases four to seven times normal when the blood supply to the tissue is blocked for a few seconds, extending to an hour or longer and then unblocked. If the block has lasted only a few seconds, this increased flow will continue for a few seconds, but if it has stopped for an hour or longer, it may continue for several hours. This condition is termed as reactive hyperemia. It arises from a local myogenic response and the vasodilators secreted due to reduced blood flow and ischemia. Reactive hyperemia is due to the release of locally generated metabolites and is part of “metabolic” blood flow regulation mechanism. It is due to defective blood flow, which initiates the cascade of events leading to vasodilatation, including tissue hypoxia and adenosine release. Following brief intervals of vascular blockage, the increased blood flow during the reactive hyperemia phase is sufficient to compensate for the tissue oxygen deficit that has developed during the blockage, almost to the exact extent of the deficit. This mechanism highlights the intimate relationship that exists between the delivery of oxygen and other nutrients to the tissues and the regulation of local blood flow [57-61].

Reactive hyperemia may indicate that long-term cardiovascular outcomes are a result of this microvascular dysfunction, as seen in cases of hypertension.

### 3.3 Active Hyperemia

**Fig. (8):**
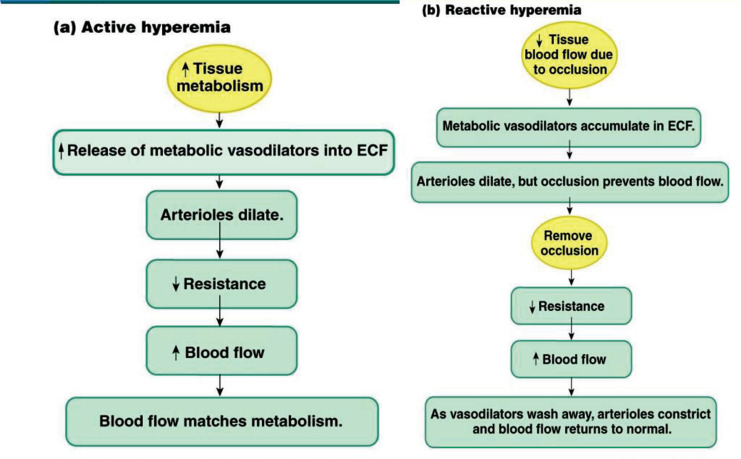
(**a, b**) Shows the mechanism of both active and reactive hyperemia. Active hyperemia is seen during active functioning of an organ while reactive hyperemia is seen when local blood flow is obstructed [61].

The rate of blood flow through any tissue increases when it becomes highly active, such as in an exercising muscle, a gastrointestinal gland during a post-prandial hypersecretory period, or even the brain during enhanced mental activity. Once more, as seen in reactive hyperemia, an increase in local metabolism leads to the cells releasing a significant amount of vasodilator substances and consuming nutrients from the tissue fluid very quickly. As a result, the local blood vessels enlarge, increasing the amount of blood flowing through them. During vigorous exercise, local muscle blood flow can increase up to 20 times due to active hyperemia in skeletal muscle (Fig. **8**).

### 3.4 Abdominal Compression Reflex

When a baroreceptor or chemoreceptor reflex is elicited, neural signals are sent simultaneously through skeletal nerves to the skeletal muscles of the body, especially the abdominal muscles. This aids in the translocation of blood from the abdominal vascular reservoirs toward the heart by compressing all of the abdomen's venous reservoirs. As a consequence, more blood is made available for the heart to pump. The “abdominal compression reflex” is the name given to this whole reaction. Both cardiac output and arterial pressure rise, the circulation is affected in the same way that sympathetic vasoconstrictor impulses constrict the veins. Given that individuals with paralyzed skeletal muscles are known to be significantly more susceptible to hypotensive episodes than those with normal skeletal muscles, the abdominal compression reflex is likely far more significant than previously thought [4, 62].

### 3.5 Autoregulation of Local Blood Flow

An acute rise in arterial pressure results in an instantaneous increase in blood flow in any tissue in the body. However, despite maintaining an elevated arterial pressure, most tissues see a return to near-normal blood flow in less than a minute. “Autoregulation of blood flow” describes this return of flow toward normal. Autoregulation is the ability of tissues to control their own blood flow. A change in arterial blood pressure is directly correlated with a change in vascular lumen diameter. The kidney, brain, heart, skeletal muscles, liver, and mesentery all have highly developed autoregulation. Two hypotheses have been proposed [4, 6, 63-66].

#### 3.5.1 Myogenic Theory of Autoregulation

The smooth muscle of the vessel wall contracts for a brief period of time when small blood vessels suddenly stretch, which is the basis for this theory. It has been suggested that reactive vascular constriction, which lowers blood flow back to normal levels, results from stretching of the vessel wall by high arterial pressure. On the other hand, the vessel stretches less at low pressures, causing the smooth muscle to relax and permit more flow.

- Vascular smooth muscle naturally exhibits the myogenic response, which can happen even in the absence of hormonal or neurological stimulation.
- Although it can be seen in arteries, venules, veins, and even lymphatic vessels, it is most noticeable in arterioles. Myogenic contraction is initiated by stretch-induced vascular depolarization, which then rapidly increases calcium ion entry from the extracellular fluid into the cells, causing them to contract.
- The theory can be explained by the law of Laplace, which states that wall tension is proportional to the distending pressure times the vessel radius. This suggests that the myogenic mechanism may play a role in preventing excessive blood vessel stretching when blood pressure is raised.

#### 3.5.2 Metabolic Theory of Autoregulation

Excess flow supplies the tissues with an excessive amount of oxygen and other nutrients when the arterial pressure rises too high. Vasodilatory substances like CO_2_, H^+^, nitric oxide, adenosine, prostaglandins, K^+^, and phosphate ions are also removed by excessive blood flow. In fact, in situations where the tissues' metabolic demands are greatly raised, such as during intense muscle exercise, results in abrupt increases in skeletal muscle blood flow. Metabolic factors seem to take precedence over the myogenic mechanism.

NOTE: Local Vasodilators: Decreased O_2_, increased CO_2_, H^+^, nitric oxide, adenosine, Prostaglandins, K^+^, increased temperature and phosphate ions

Local Vasoconstrictors: O_2_, epinephrine, norepinephrine, serotonin, decreased temperature.

### 3.6 Sino-aortic or Marey’s Reflex (Cardioinhibitory Reflex)

**Fig. (9):**
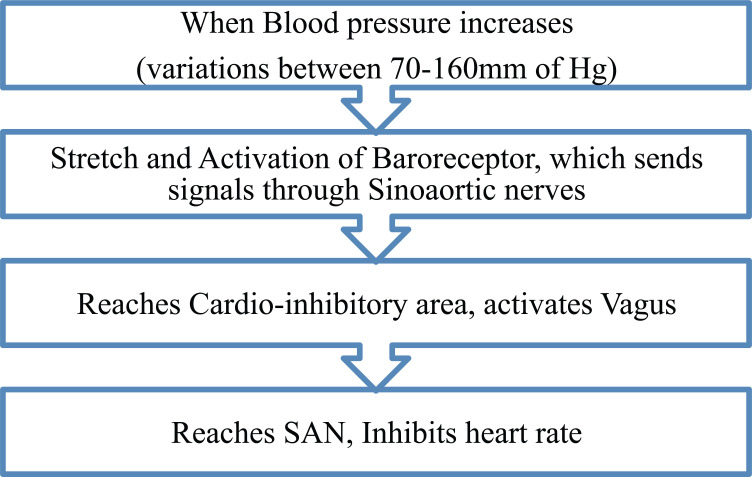
Flow chart for Marys reflex. Fall in blood pressure, causes an inverse effect through inhibition of cardio-inhibitory area, in-turn inhibiting vagus.

Acute changes in the blood pressure reflexly, proportionally and inversely change heart rate. This occurs *via* the baroreceptors. This effect is seen over intermediate range of blood pressure (between 70-160 mm of Hg). Below this range, heart rate is constantly high while above the range, heart rate is constantly low. Sino-aortic nerves supplying the carotid sinus and aortic arch get activated by variations in blood pressure. When blood pressure rises, the sino-aortic nerves increase vagal tone by activating the cardiac-inhibitory area. A fall in blood pressure increases heart rate by increasing the sympathetic discharge. This reflex is activated only in physiological conditions. Exercise, emotional stress, anoxia and so on increase both blood pressure and heart rate (Fig. **9**) [2, 3, 67, 68].

### 3.7 Oculocardiac Reflex (Aschner Reflex or Trigeminovagal Reflex (TVR)

The first account of it dates back to 1908, when direct pressure applied to the eyeball led to a decrease in heart rate. A heart rate drop of more than 20% results in sinus bradycardia. On the other hand, it has also been linked to asystole, arrhythmia, decreased arterial pressure, and even cardiac arrest. The most prominent examples of this reflex are those occurring during ophthalmologic procedures that result in arrhythmia, decreased atrial pressure, ventricular tachycardia, ventricular fibrillation, and other complications. The immediate removal of the triggering stimulus is the definitive course of treatment [69-71].

Stretch receptors in the ocular and periorbital region start the pathway for this reflex, which continues to the ciliary ganglion, the trigeminal nerve's ophthalmic division, the Gasserian ganglion, the trigeminal nucleus, and finally the nucleus tractus solitarious, where the afferent limb ends. The trigeminal sensory nucleus and the visceral motor nucleus of the vagus nerve communicate with each other intranuclearly. Bradycardia is caused by the vagus nerve inhibiting the SA node.

Afferent: Trigeminal nerve activated by pressure on the eye globe.

Processor: Sensory nucleus of V cranial nerve; and NTS.

Efferent: Vagus nerve along with sympathetic chain

**Fig. (10):**
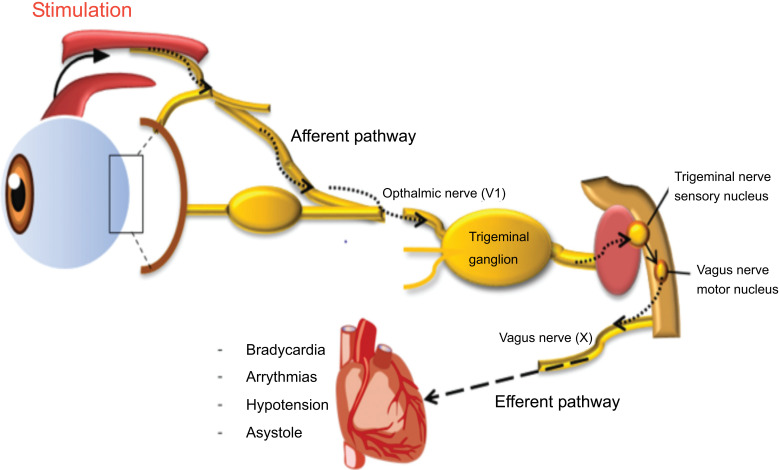
[71]: Diagrammatic representation of the oculocardiac reflex. It depicts the interaction of vagus with trigeminal nerves.

Effect: Cerebral vasodilation, vagal bradycardia, systemic vasoconstriction (Fig. **10**).

### 3.8 Diving Reflex

When we hold our breath and submerge in water, the face and nose become wet, which causes bradycardia, apnoea and increased peripheral vascular resistance, these together form the diving reflex. Increased vascular resistance is to redistribute blood to vital organs. Bradycardia limits unnecessary peripheral oxygen consumption. This reflex is present in all vertebrates that work to preserve oxygen stores. This can be used as an effective means to treat paroxysmal supraventricular tachycardia. Diving reflex can be elicited by using a cold application on the face to increase vagal tone [72-75].

The nerve fibers supply anterior nasal mucosa and paranasal region. The trigeminal afferents relay to the brain stem and activate sympathetic signaling to blood vessels, increasing peripheral vasoconstriction. Also, the parasympathetic system of the heart induces bradycardia. When divers hold breath, blood gas variations activate the chemoreceptors which further enhances peripheral resistance ensuring adequate oxygen stores to vital organs.

Sudden infant death syndrome (SIDS), the probable hypothesis revolves around hyperactive dive reflex in infants, which might explain the pathophysiology of apnea, bradycardia and increased peripheral resistance leading to SIDS. Dive reflex can be used to treat paroxysmal supraventricular tachycardia (PSVT).

Afferent: Trigeminal nerve activated by cold temperature; pressure of immersion.

Processor: Sensory nucleus of V cranial nerve; and NTS.

Efferent: Vagus nerve along with sympathetic chain

**Fig. (11):**
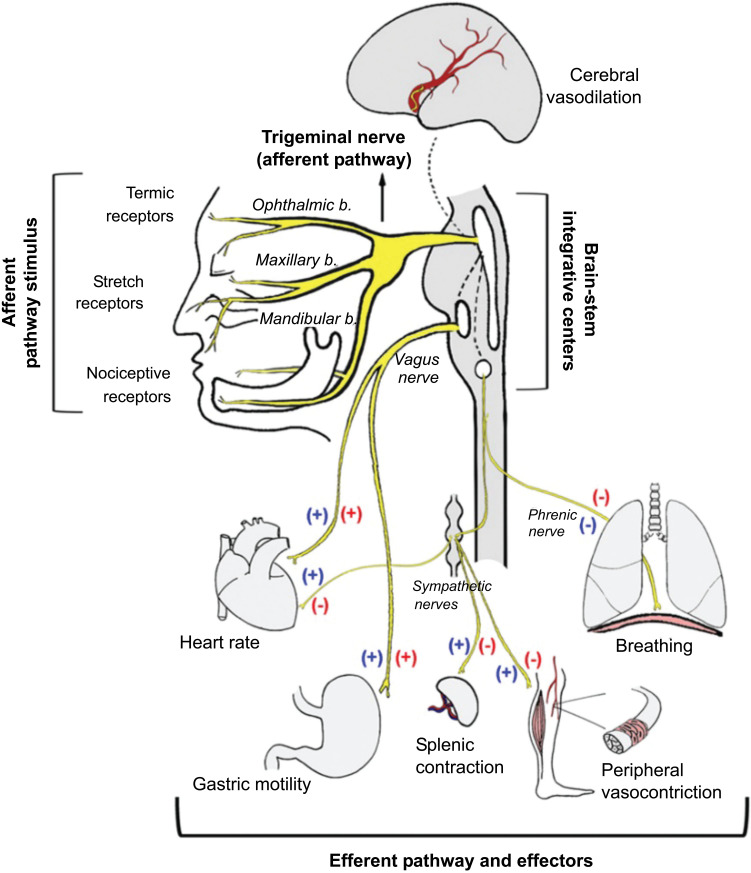
Protective reflexes includes the diving, the nasopharyngeal, or the oculocardiac reflex. Simultaneous activation of both sympathetic and parasympathetic limbs is seen (blue and red symbols) [73].

Effect: Cerebral vasodilation, vagal bradycardia, systemic vasoconstriction (Fig. **11**).

### 3.9 Valsalva Response

This response assesses the integrity of the physiological response to baroreceptor activation and its control over heart rate and blood pressure. Normally, an increase in blood pressure reduces heart rate *via* vagus, while a decrease in blood pressure increases heart rate by decreasing vagal activation. In supine position, the subject is asked to exhale against a closed glottis (by keeping the nose and lips tightly closed and maintaining a pressure of 40mm Hg into a manometer) for 15 sec. heart rate and blood pressure are recorded. 4 phases are observed [76-81].

- *Phase I*: Blood pressure rises and heart rate falls – mechanical response seen due to increased intrathoracic and intra-abdominal pressure.
- *Phase II:* Blood pressure falls and heart rate rises – seen due to reduced venous return which activates baroreceptor reflex, causing tachycardia and increased peripheral resistance (this arrests BP drop within 5-8 sec after onset on maneuver. The first part of response requires reduction in vagal tone while 2^nd^ part requires an intact sympathetic nervous system).
- *Phase III:* Blood pressure falls and heart rate rises – this phase is seen at the end of maneuver, and is a mechanical response like phase I.
- *Phase IV:* Blood pressure rises and heart rate falls – caused by vasoconstriction and adrenergic induced blood pressure overshoot and bradycardia persists and is mediated by baroreceptor.

This reflex is seen while straining at stools, in trumpet players, and in heavy weight lifters and is useful in clearing earblocks during flight descent. Historically, it has been tried in draining brain abscesses, intracranial and middle ear fluids and head injury patients. Valsalva induced blackouts and convulsions have also been noted.

**Fig. (12):**
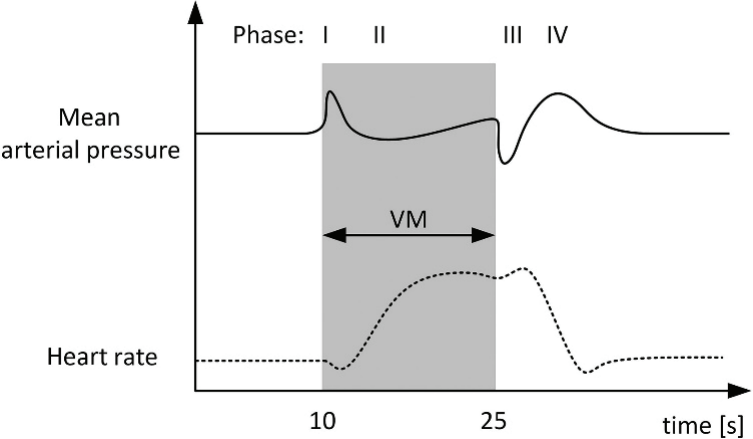
Shows 4 phases of Valsalva response [80].

Modified Valsalva maneuver [81] is also developed, which overcomes the draw backs of standard Valsalva. Modified version requires the patient to lie in supine position with legs raised for 45 sec after performing the standard Valsalva (Fig. **12**).

## 4 CARDIOPROTECTIVE ROLE OF VAGUS DURING REPERFUSION OF ISCHEMIC CARDIAC TISSUE

The concept of Remote ischemic conditioning (RIC) points to the idea that short, reversible stimulus of ischemia followed by reperfusion in one part of the vascular bed or organ, provides protection and far-off organ resistance to ischemic injuries. An example of RIC is repeated inflation and deflation of BP cuff on a limb, gives additional protection to repercussed acute myocardial infarct tissue. Both neural and humoral mechanisms have been hypothesized to be involved. Efferent vagus nerve is thought to play a significant role, though the entire mechanism needs further research [82-87].

Afferent: Stimulus originates from local ischemia/reperfusion injury like mesentery or limb, also from local surgical trauma, local capsaicin activation of sensory fibers, bradykinin, or adenosine, local electrical nerve stimulation, local anesthesia with lidocaine, or a sensory nerve blocker and transection of the peripheral nerve. They seem to activate the spinal reflexes.

Efferent: Through autonomic nervous system, activates cardiac vagal nerves and also releases cardioprotective substances through sympathetic efferents Figs. (**13** and **14**).

## 5 CAROTID SINUS MASSAGE

As mentioned earlier, the carotid sinus is a part of barorceptors that has sensory nerve fibers running along the entire adventitia of the first segment of internal carotid artery, up-till the bifurcation of carotids. Carotid massage releases excitatory signals from baroreceptors, which, *via* the central mechanisms, decreases blood pressure and heart rate. This forms a part of “Vagal Maneuvers” which are used to increase parasympathetic tone and block AV nodal transmission. “Vagal maneuvers” include carotid sinus massage, standard Valsalva, modified Valsalva, and diving reflex, to name a few [88-92].

Carotid sinus massage has both diagnostic and prognostic values. It is indicated to assess the cause of syncope in patients > 40 years’ and also as a treatment for supraventricular tachycardia. 3 possible responses are seen with carotid massage: the cardioinhibitory response, vasodepressor response and mixed response. Characteristic of the response helps in guiding for further clinical management.

Carotid sinus reestablishes sinus rhythm in patients with supra ventricular tachycardia. It also helps in identifying abnormal atrial rhythms by slowing ventricular rhythms, which allows the diagnosis of atrial flutter.

## 6 ROLE OF VAGUS ALONG WITH BARORECEPTOR AS TREATMENT MODALITY IN HEART FAILURE PATIENTS

Heart failure is generally accompanied with autonomic dysfunction, (with sympathetic over activity and vagal withdrawal), along with reduced ejection fraction. Probable mechanism is the failure of mechanical stretch of baroreceptor that accompanies defective left ventricular function. A sudden disruption in vagal fibers and over activation of sympathetic nerves sets into motion. Correcting the autonomic imbalance can rectify the malfunction mechanisms in heart failure patients [93-96].

**Fig. (15):**
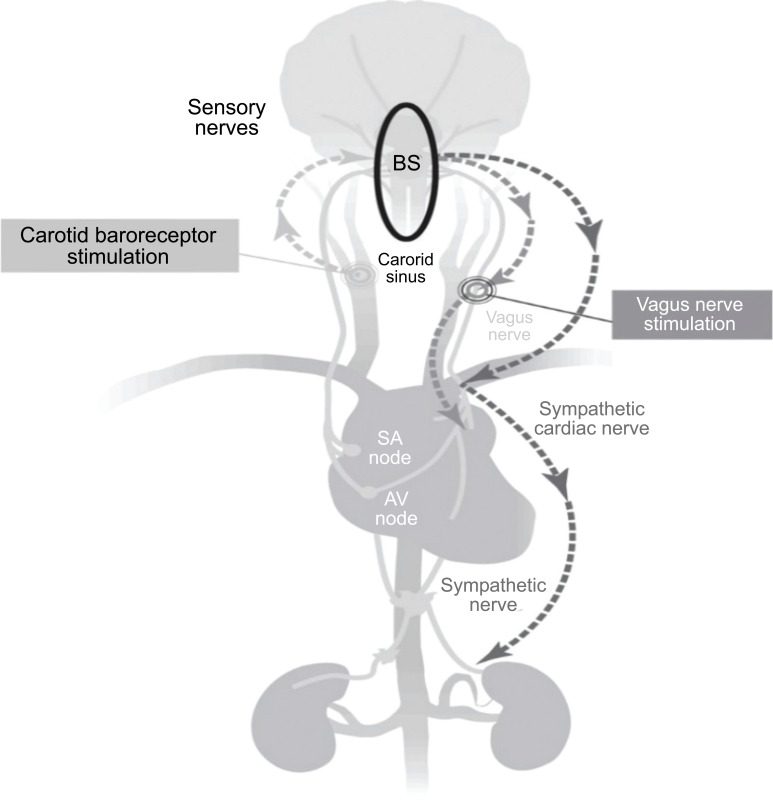
With myocardial infarction, baroreceptor dysfunction also ensues. Hence direct activation of vagus can curb MI symptoms [95].

In this regard, renal sympathetic denervation, vagal nerve stimulation and baroreceptor activation therapy are under evaluation. Of these, baroreceptor activation therapy has shown promising results in heart failure patients who are not suitable for cardiac resynchronization therapy. Baroreceptor activation is shown to improve left ventricular function and also is seen to revert ventricular remodeling, both globally and microscopically. Direct stimulation of the vagus nerve, independent of the afferent fibers to CNS helps curb heart failure symptoms (Fig. **15**).

## 7 VAGAL ABLATION AS THE TREATMENT OF CHOICE

**Fig. (16):**
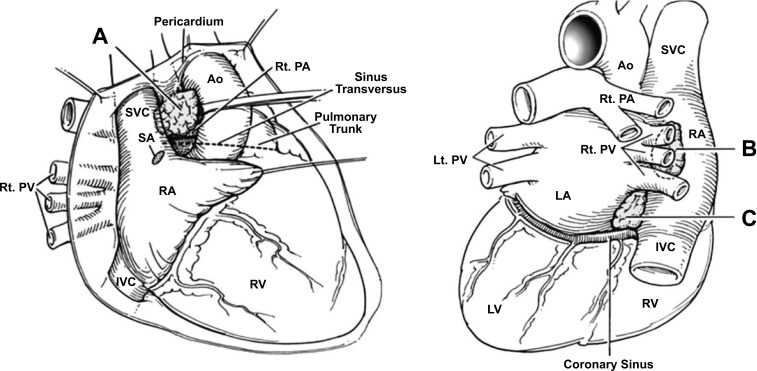
Showing 3 ganglias identified for radiofrequency ablation as treatment modality for bradyarrhythmias [100].

Functional bradyarrhythmias are a set of transiant disorders which present with vagal predominance on the cardiac pacemaker, reduced sympathetic tone and a lack of organic cause. These include AV nodal block, sinus dysfunction, cardioinhibitory neurocardiogenic syncope, carotid sinus syndrome, *etc*. These affect the quality of life of the patients, causing weight loss, dizziness and syncope. The dorsal nuclei of vagus within medulla sends preganglionic fibers mainly to atria, which reduces automaticity, conductivity and excitability. 3 parasympathetic ganglias are located within atrial wall of which Ganglia B is chosen to achieve parasympathetic ablation (Fig. **16**) [96-100].

## CONCLUSION

The paired 10th cranial nerve, also called the Vagus or Wanderer nerve, forms an important innervation for many viscera’s and organ systems. This article aimed at showcasing the role of vagus as a key component, either as afferent or efferent or both in regulating few important reflexes which control the cardiovascular system. These reflexes include a physiological response, a pathological consequence or a clinical correlate.

## Figures and Tables

**Fig. (13):**
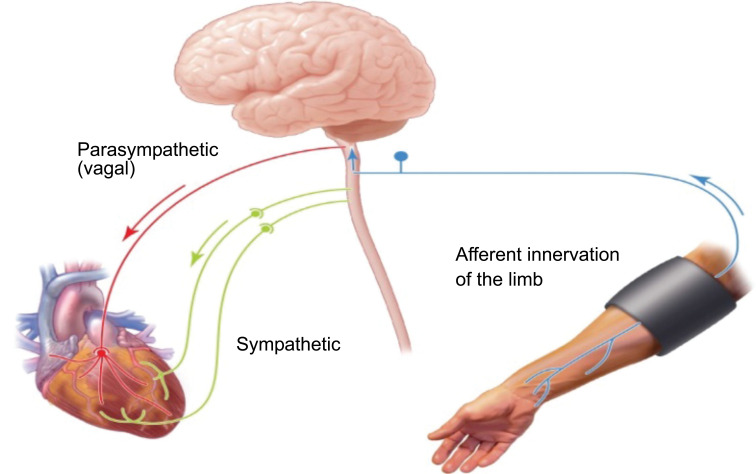
Shows reflex arc of Remote ischemic cardioprotection. This reflex is hypothesized to be comprised of C-fiber sensory innervation of the remote ischemic organ/tissue and vagal innervation of the heart. It may also include sympathetic innervation [82] (Fig. **14**).

**Fig. (14):**
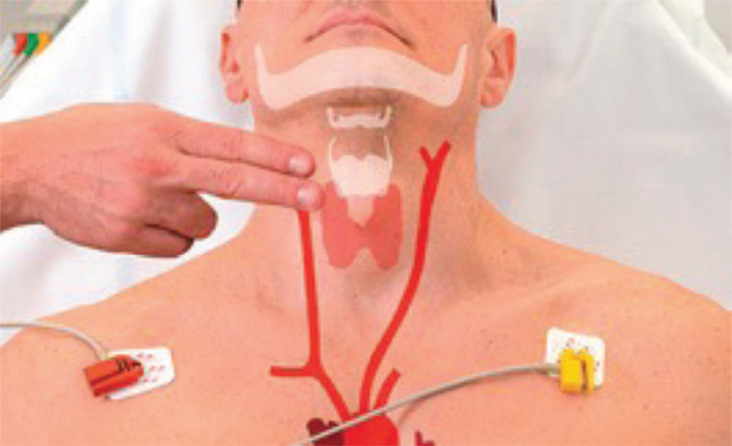
Showing location of carotid sinus for carotid sinus massage [88].

## LIST OF ABBREVIATIONS

MAP: Mean Arterial Blood Pressure
NTS: Nucleus Tractus Solitarius
ICP: Intracranial Pressure
